# Seoul Virus Tropism and Pathology in Naturally Infected Feeder Rats

**DOI:** 10.3390/v11060531

**Published:** 2019-06-07

**Authors:** Miriam Maas, Melanie van Heteren, Ankje de Vries, Thijs Kuiken, Tabitha Hoornweg, Edwin Veldhuis Kroeze, Barry Rockx

**Affiliations:** 1 Center for Infectious Disease Control, National Institute for Public Health and the Environment, 3720 BA Bilthoven, The Netherlands; Ankje.de.Vries@rivm.nl (A.d.V.); tabitha.hoornweg@rivm.nl (T.H.); 2 Department of Viroscience, Erasmus University Medical Center, 3015 GD Rotterdam, The Netherlands; m.vanheteren-hemelop@erasmusmc.nl (M.v.H.); t.kuiken@erasmusmc.nl (T.K.); e.veldhuiskroeze@erasmusmc.nl (E.V.K.)

**Keywords:** Seoul virus, reservoir, tropism, inflammation

## Abstract

Seoul virus (SEOV) is a zoonotic orthohantavirus carried by black and brown rats, and can cause hemorrhagic fever with renal syndrome in humans. Human cases of SEOV virus infection have most recently been reported in the USA, United Kingdom, France and the Netherlands and were primarily associated with contact with pet rats and feeder rats. Infection of rats results in an asymptomatic but persistent infection. Little is known about the cell tropism of SEOV in its reservoir and most available data is based on experimental infection studies in which rats were inoculated via a route which does not recapitulate virus transmission in nature. Here we report the histopathological analysis of SEOV cell tropism in key target organs following natural infection of a cohort of feeder rats, comprising 19 adults and 11 juveniles. All adult rats in this study were positive for SEOV specific antibodies and viral RNA in their tissues. One juvenile rat was seropositive, but negative in the rRT-PCR. Of the 19 adult rats of which subsequently additional organs were tested, SEOV RNA was detected in all lungs, followed by kidney (79%) and liver (74%). Histopathologic changes associated with SEOV infection were primarily found in the liver, consistent with a pathological diagnosis of a mild hepatitis. In conclusion, natural SEOV infection results in mild inflammation of the liver in the absence of clinical disease.

## 1. Introduction

Seoul virus (SEOV) is an emerging zoonotic virus (genus *Orthohantavirus*, family *Hantaviridae*) that may cause hemorrhagic fever with renal syndrome (HFRS) in humans [1,2]. SEOV is associated with brown (*Rattus norvegicus*) and black rats (*Rattus rattus*) as its reservoir and is found worldwide due to the geographic distribution of these rats [3]. Orthohantaviruses are usually transmitted from rodents to humans via inhalation of contaminated fomites, but can also be transmitted through bites [4]. While the respiratory route is considered the main route of transmission from rats to humans, transmission between rats has also been associated with wounding through biting and/or scratching [5]. Recent human cases of autochthonous SEOV infection in the USA, United Kingdom, France and the Netherlands were primarily associated with contact with pet rats and feeder rats [6,7,8,9]. Although SEOV has been detected in wild rats in the Netherlands [10], the spread is likely limited [11]. Human cases due to exposure to wild rats have been extensively described in Asia, however the risk of SEOV from wild rats in the Netherlands seems less prominent [12].

Interestingly, while orthohantavirus infection in humans can lead to severe disease, infection in the reservoir species is considered asymptomatic and persistent [13,14,15]. Most information about the pathology of SEOV in rats and the immune response has been acquired through experimental infection [14]. Limited information is available on SEOV cell tropism, and in agreement with other orthohantaviruses, endothelial cells are an important target in these studies [16]. However, most experimental infection studies have focused on intraperitoneal inoculation as the route of experimental infection [14]. Limited data is available on the natural history of SEOV infection in rats. Previous studies with other viruses such as influenza and henipaviruses have shown that the route of inoculation may affect cell tropism and therefore it is important to compare experimental data with data acquired through natural infections [17,18]. Here we studied the tissue and cell tropism of SEOV in a cohort of naturally infected pet rats.

## 2. Materials and Methods

### 2.1. Ethics Statement

The animals were collected by the Netherlands Food and Consumer Product Safety Authority and tested by the National Institute for Public Health and the Environment as part of source finding related to a human SEOV patient [6].

### 2.2. Animal Studies

Sixty rats (*Rattus norvegicus*) were collected from a feeder farm linked to a human case of SEOV infection previously described [6]. Forty animals were adults (age 7–13 months) and 20 were juveniles (age 4–6 weeks). Animals were euthanized by means of exsanguinations under sedation. Serum was collected for serology, and whole-blood samples (EDTA Vacutainer), urine, oral and rectal swabs as well as lung, trachea, kidney, spleen, urinary bladder, liver, salivary gland, pancreas, stomach, small and large intestines were sampled for RNA isolation, and histological examination by a licenced veterinary pathologist.

### 2.3. Detection of SEOV Antibodies

Antibodies in rat serum were detected by using a human SEOV ELISA (Hantavirus Dobrava/Hantaan IgG Elisa; Progen Biotechnik GmbH, Heidelberg, Germany), which was adapted to enable detection of immunoglobulin G (IgG) in rats. Rabbit-α-rat horseradish peroxidase-labeled IgG (Sigma-Aldrich Chemie B.V., Zwijndrecht, the Netherlands) was used as conjugate at a 1:5000 dilution. A cut off value was based on the average optical density (OD) of negative control rat serum + 3× SD (in this case, a value of 0.2–0.3) as described previously [6].

### 2.4. Detection of Viral RNA

Lung, kidney and liver tissues were collected in RNAlater (Applied Biosystems, Foster City, CA, USA) and stored at −80 °C. Tissue samples (+/− 25 mg per sample) were disrupted in MagNA Pure 96 External Lysis Buffer (Roche) by using Lysis matrix D (MP Biomedicals, Santa Ana, CA, USA) and Fast Prep FP120 Homogenizer (Thermo Savant, Carlsbad, CA, USA). RNA isolation was performed on blood (200 µL), urine (200 µL), saliva and rectal swabs using a QIAamp Viral RNA kit. All samples were tested by a SEOV–specific real-time RT-PCR as previously described [6]. Equine Arteritis Virus was added in the lysis buffer to all samples prior to nucleic acid extraction to exclude inhibitory factors in the sample itself as quality assurance for each experimental step [19]. A rat β-actin control was used as an amplification control by conventional RT-PCR, to confirm the extraction of nucleic acid from tissue material. Only animals with samples positive for β-actin were included.

### 2.5. Pathology & Histology:

All tissue samples were immersion-fixed in 10% neutral buffered formalin solution for at least 7 days. For a random selection of animals (19 adult, 11 juvenile animals), formalin-fixed samples of lung, kidney, spleen, and liver, were routinely processed, paraffin embedded, and cut to 3–4 μm on glass slides, deparaffinized with xylene and rehydrated using graded alcohols, and stained with haematoxylin & eosin (HE) for histopathological examination by light microscopy. Serial sections were simultaneously cut and stained for the presence of SEOV nucleoprotein (N protein) by immunohistochemistry (IHC) using a mouse anti-SEOV N protein monoclonal antibody at a dilution of 1:400 (kindly provided by Dr. Indre Kucinskaite-Kodze, Institute of Biotechnology of Vilnius University) [20], and visualized by using a goat anti-mouse horseradish peroxidase followed by aminoethyl carbazole and a haematoxylin counterstain. Several serial sections of representative tissues were additionally cut and stained for IHC with an antibody against CD31 (RnD systems) to identify vascular endothelial cells in tissues.

H&E sections were evaluated for any histopathological changes, including inflammatory reactions or other lesions. Because infiltration with polymorphonuclear cells (PMNs) was noted upon screening of SEOV-positive tissues, semiquantitative assessment of PMN infiltration in the lung, kidney and liver was performed using a method modified from previous reports [21,22]. Briefly, liver and lung tissues were scored randomly and blinded for the number of polymorphonuclear leukocytes (PMN) within the parenchyma excluding large blood vessels. In 5 randomly selected microscopic high-power fields (HPF; 400× magnification field of an Olympus BX51 light microscope) the total number of cells were counted and presented as the number of PMNs/5HPF. Furthermore, kidney slides were scored similarly for the number of PMNs in 25 randomly selected glomeruli per animal and expressed as number of PMNs/25 glomeruli. PMNs entail both neutrophilic granulocytes and eosinophilic granulocytes, of which neutrophils were most abundant.

### 2.6. Statistics

Comparisons of pathology scores were performed by using Student’s *t*-tests and found statistically significant if *p* < 0.05. All data are presented in figures as means ± sd.

## 3. Results

### 3.1. Clinical Signs

In general, rats had a good body condition score, but many animals had a poor coat condition, ear lesions suggestive for mites, as well as bite wounds. Four juveniles died from unknown reasons before they could be euthanized and were excluded from the study. Ten females were pregnant.

### 3.2. Laboratory Findings

As previously described, only 1 out of 16 juvenile rats was seropositive whereas all adult rats tested positive for orthohantavirus IgG antibodies [6]. A random selection of 19 adult rats, and 11 juveniles (1/11 seropositive), was tested for the presence of SEOV RNA by rRT-PCR, in different biological samples. Of the adult rats, SEOV RNA could primarily be detected in the lungs of all adult rats (19/19), but also in the kidneys in 79% (15/19), liver in 74% (14/19) and the blood in 74% (14/19) of the rats. To a lesser extent, SEOV could be detected in the saliva swabs 63% (12/19), rectal swabs 42% (8/19) and urine 16% (3/19) (Table 1). These data suggest that persistent infection occurs primarily in the lung and that viral shedding is limited in the number of animals. The 11 juvenile rats tested negative by rRT-PCR in all samples, including the seropositive animal.

### 3.3. Pathological Findings Rats

#### 3.3.1. Gross Lesions

Five adult animals showed lesions varying from enlarged bronchial lymph nodes (*n* = 2), enlarged spleen (*n* = 1), petechiae on the salivary gland (*n* = 1) to penile prolapse, empty gastrointestinal tract and blood from nose during anesthesia (*n* = 1). Of the juveniles, 14/16 showed enlarged spleens.

#### 3.3.2. Histopathology in Lungs

**Figure 1:**
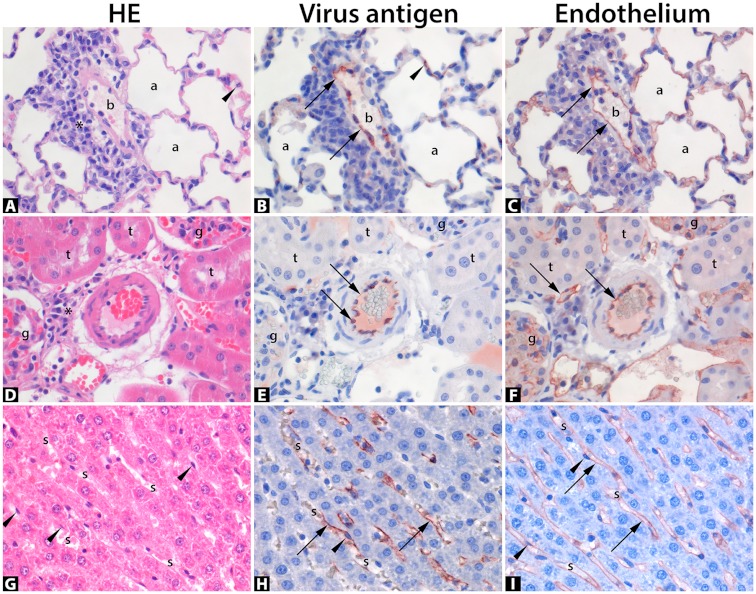
Histopathology of natural Seoul virus (SEOV) infection in feeder rats. Panel photomicrographs of lung (**A**–**C**), kidney (**D**–**F**), and liver (**G**–**I**) of naturally infected rats with Seoul virus, stained with haematoxylin & eosin (HE; (**A**,**D**,**G**)), or by immunohistochemistry for virus antigen (**B**,**E**,**H**), or for endothelial cells (**C**,**F**,**I**). Positive antigen expression is visualized as finely-granular reddish-brown staining by AEC-immunoperoxidase, on Haematoxylin counterstain. Original magnifications 400×. (**A**) Lung parenchyma shows a blood vessel (b) with a mild perivascular lymphoplasmacytic infiltrate (asterisk) and surrounding air-filled alveoli (a) with a polymorphonuclear leukocyte (arrowhead) in an alveolar septum; compared to serial section (**B**) showing SEOV-antigen expression within the flattened cytoplasm of endothelial cells (arrows) lining the blood vessel lumen (b) as well as lining capillaries within alveolar septa (arrowhead); compared to serial section (**C**) corroborating virus infection in endothelial cells by positive CD31-antigen expression (arrows) specific for endothelial cells. (**D**) Kidney parenchyma centrally shows a cross-section of a thick-walled arteriole, partly filled with erythrocytes, with a mild juxtavascular lymphoplasmacytic infiltrate (asterisk) and surrounded by several renal tubules (t) and two glomeruli (g); compared to serial section (**E**) showing positive endothelial cells’ cytoplasm for SEOV-antigen (arrows), compared to serial section (**F**) corroborating endothelial cells by positive CD31-antigen expression (arrows). (**G**) Liver parenchyma diagonally shows narrow sinusoids (s) in between hepatocellular cords. The sinusoids contain few erythrocytes and are lined by endothelial cells with nuclei slightly bulging into the sinusoidal lumens (arrowheads); compared to section (**H**) * showing abundant positive expression of the endothelial cell cytoplasm for SEOV-antigen (arrows); compared to serial section (**I**) corroborating endothelial cells by positive CD31-antigen expression (arrows). * Photomicrograph H pertains a representative liver section, not an exact serial section.

The lungs and airways including tracheas showed neither marked inflammation nor SEOV-IHC positive epithelial cells. Lymphoplasmacytic aggregates of mild cellularity were present in the interstitia around blood vessels and airways of several but not all infected animals (Figure 1A), therefore without apparent correlation to viral infection of the lungs or the animal. Several lungs showed extensive granular cytoplasmic positive IHC staining for SEOV of especially interstitial endothelial cells of alveolar septal capillaries, and seldom of endothelial cells lining larger blood vessels such as pulmonary veins (Figure 1B). The endothelial cell origin of the infected cells was corroborated by additional IHC staining of serial lung slides with an antibody against CD31—a marker for endothelial cells (Figure 1C).

**Figure 2:**
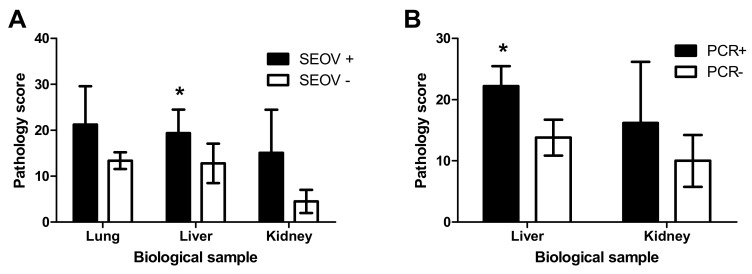
Pathology scoring in lung liver and kidney of SEOV infected rats. A comparison of histological scoring of samples stained with haematoxylin & eosin (HE) was performed between adult animals that were seropositive (SEOV +) and juvenile animals that were seronegative (SEOV −; (**A**)), or in adult animals that were positive (PCR +) or negative (PCR−; (**B**)) for viral RNA in liver or kidney. Bars present the average score between animals. The error bars represent the standard deviation of the mean (* = *p* < 0.05, Student’s *t*-test).

The number of PMNs present within the pulmonary alveolar interstitial tissues were counted and compared with viral infection (Figure 2A). While there was a trend of a higher number of PMN in lungs of SEOV positive adult animals compared to SEOV negative juvenile animals, this was not statistically significant (Figure 2; *t*-test, *p* = 0.053) and was confounded by the age difference between. Since all of the lungs of adult animals was positive for SEOV by RT-PCR, a comparison of age-matched SEOV positive and negative animals from the same population was not possible.

#### 3.3.3. Histopathology in Kidneys

The kidneys showed neither marked inflammatory lesions nor SEOV IHC positive epithelial cells. Few lymphoplasmacytic aggregates of mild cellularity were present within the renal interstitium of few animals (Figure 1D), therefore without apparent correlation to viral infection of the kidneys or the animal. Capillaries of these organs showed granular cytoplasmic positive SEOV IHC staining of variable extent. Again, seldom endothelial cells lining larger blood vessels such as arterioles of kidneys were found positive for SEOV by IHC (Figure 1E). The endothelial cell origin of the infected cells was corroborated by additional IHC staining of representative kidney tissues with an antibody against CD31 a marker for endothelial cells (Figure 1F). In accordance with lung samples, the number of PMNs present within 25 renal glomeruli per animal were counted and compared with infection status (Figure 2A) and presence of viral RNA and no significant difference was observed (Figure 2B).

#### 3.3.4. Histopathology in Liver

**Figure 3:**
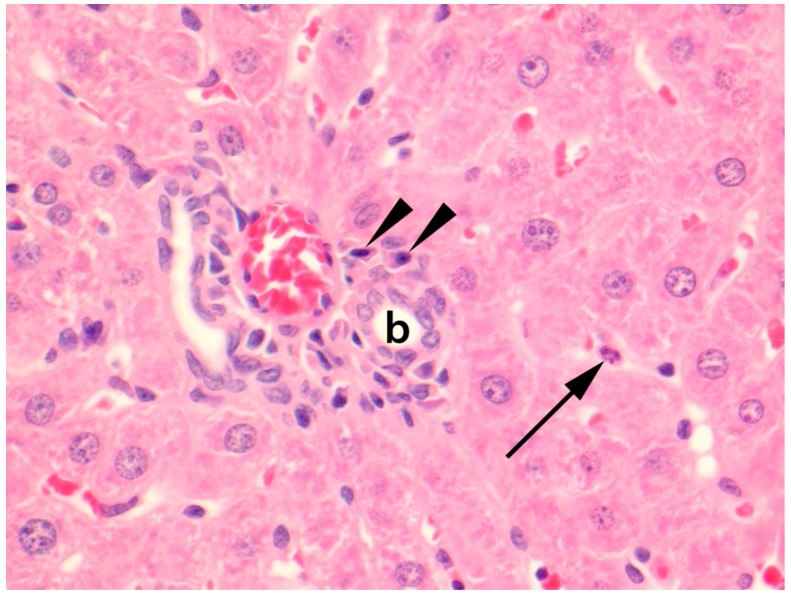
Photomicrograph of liver parenchyma of a natural Seoul virus (SEOV) infection in a feeder rat. A polymorphonuclear leukocyte (arrow) is present within a hepatic sinusoid, and centrally, a portal area with a bile duct (**b**) and blood vessels contains a mild lymphoplasmacytic aggregate of which plasma cells (arrowheads) are evident. Stained with haematoxylin & eosin (HE). Original magnification 400×.

A consistent finding of the liver tissues was mild hypercellularity within the hepatic sinusoids mostly comprised of PMN (Figure 3).

Furthermore, lymphoplasmacytic aggregates of mild to moderate cellularity were present in hepatic portal areas and surrounding central veins of several but not all infected animals, therefore without apparent correlation to viral infection of the liver or the animal. Several livers showed granular cytoplasmic positive IHC staining of especially endothelial cells lining the sinusoids (Figure 1H), and seldom of endothelial cells lining larger blood vessels such as portal veins or hepatic arteries. The endothelial cell origin of the infected cells was corroborated by additional IHC staining of representative liver tissues with an antibody against CD31 a marker for endothelial cells (Figure 1I). The number of PMNs present within the hepatic parenchyma and sinusoids were significantly higher in SEOV infected animals compared to non-infected (Figure 2A; *t*-test, *p* = 0.018). However since, there is a difference in age between the two groups, we also performed analysis between adult animals that were positive for SEOV RNA in liver versus those that were negative and found a highly significant correlation between the number of PMN and SEOV infection in the liver by RT-PCR (Figure 2B; *t*-test, *p* < 0.001). These data show that persistent SEOV infection of the liver in rats primarily targets the microvasculature and results in mild yet obvious inflammation, consistent with a pathological diagnosis of a mild hepatitis.

#### 3.3.5. Other Histopathological Findings

Urinary bladders, salivary glands, gastro-intestinal tracts, pancreases, spleens, showed neither marked inflammatory lesions nor SEOV IHC positive epithelial cells. Capillaries of these organs showed granular cytoplasmic positive SEOV IHC staining of variable extent.

## 4. Discussion

SEOV is a rodent-borne zoonotic virus that may cause severe disease in humans, but results in an asymptomatic but persistent infection in rats. Little is known about the cell tropism of SEOV in its reservoir and most available data is based on experimental infection studies in which rats were inoculated via a route which does not recapitulate virus transmission in nature. Here we report the histopathological analysis of SEOV cell tropism in key target organs following natural infection of a cohort of feeder rats associated with a human case of SEOV infection in the Netherlands [6].

Interestingly, all adult rats in this study were positive for SEOV specific antibodies and viral RNA in their tissues. The rats in this study were obtained from a feeder rat breeding farm. They had been housed in open boxes, and in close quarters, with 5–10 male and 20–25 female rats per box. The observed poor coat condition, ear lesions and bite wounds are considered conductive for animal-to-animal transmission [5], and would explain the high prevalence of the virus in this population. A similar high prevalence of 100% infection in rats has previously been reported in breeding colonies in the UK [23]. Surprisingly, one juvenile rat which was positive for orthohantavirus IgG, did not have detectable levels of SEOV RNA in any tissues tested. This finding may reflect a past infection that was resolved, however since SEOV infection is generally believed to result in a persistent infection, it is more likely that the levels of SEOV RNA in these tissues are below the level of detection, as has been reported in experimental infection models [24].

In adults, of all tissues that were positive for SEOV RNA, the lung was the preferred organ for the detection of SEOV in rats, followed by liver and kidney. However, since we do not know when exactly the animals got infected, we cannot exclude that the observed differences in the number of infected organs is perhaps related to the time since infection. Lung and kidney are often used as the target organs for molecular detection of orthohantaviruses in surveillance studies [11,23,25,26]. Furthermore, SEOV RNA was more often detected in saliva swabs than in rectal swabs or urine. And while infectivity of the virus in saliva was not tested in this study, this supports previous studies that biting is an important transmission route between rats [5]. However, no limit of detection has been established for the used method, and the starting volumes and quantities of the material for the swabs and urine differed, leaving the possibility for false- negative results, which complicates a real comparison of these samples.

Interestingly, in experimentally infected rats, SEOV infection causes subclinical, acute, and focal hemorrhage and edema in the lungs [27]. In the current study, only mild inflammation was observed in lungs of some SEOV infected adult rats but not in uninfected juvenile rats. However, the differences in inflammation were not significant and more likely due to differences in age and thus prolonged exposure to environmental agents as all SEOV positive animals were adults, while all negative animals were juveniles. This observed difference in severity of histopathological changes in the lungs could be due to the route of infection. In the experimental studies, rats were inoculated via the intraperitoneal route, and while the exact route of transmission in these feeder rats cannot be determined, the most likely routes would be through wounding and/or respiratory transmission. Previous studies with other viruses such as influenza and henipaviruses have already shown that the route of transmission can impact the tropism and severity of disease [17,18]. Alternatively, hemorrhage has only been reported in lungs of rat up to 30 days post infection, and no data is available from later time points. Since we do not know for how long the rats from the breeding farm have been infected, it is possible that the rats from this study exhibited similar lesions earlier during the infection, and have since resolved the inflammation.

In the current study, histopathologic changes associated with SEOV infection were primarily found in the liver. Limited information on SEOV infection in the liver of reservoir species has been reported, with studies mostly focussing on lung, kidney and spleen [27]. Interestingly, several studies have also reported liver involvement following SEOV infection in humans. This included acute viral hepatitis-like manifestations with lobular necrosis without viral inclusions, atypical cells, vasculitis, or fibrosis, a painful enlarged liver and distinct elevation of liver enzymes [6,28,29,30]. This suggests that SEOV may have a tropism for liver in both reservoir and diseased host. The mechanisms of pathogenesis in the liver are the focus of ongoing studies.

Overall, in all tissues evaluated, SEOV primarily targeted endothelial cells lining the microvasculature and seldom endothelial cells lining larger blood vessels. This is in line with previous reports for SEOV and other orthohantaviruses [31,32,33]. In humans, orthohantavirus infection of the endothelial cells can result in increased permeability and hemostatic dysfunction [34,35], however the effect of infection on endothelial cells in reservoir species has not been studied in detail.

In conclusion, SEOV causes a persistent infection in different tissues in rats, primarily targeting the endothelial cells. With both experimental and natural infection, SEOV infections result in a persistent infection in the absence of clinical disease. Infection in the liver can lead to mild inflammation. This suggests that an infection with SEOV does cause an immune response in rats, but that the effects of this are rather limited and often go unnoticed. These results further highlight the importance of mimicking the natural route of infection for further studies into the pathogenesis of SEOV infection in its reservoir host.

## Figures and Tables

**Table 1:** Results of the rRT-PCR of 30 rats: 20 seropositive rats, including 1 juvenile, and 10 seronegative rats.

		number tested	lung (pos)	kidney (pos)	liver (pos)	blood (pos)	saliva swab (pos)	rectal swab (pos)	urine (pos)
**seropositive**	**adult**	19	19	15	14	14	12	8	3
	**juvenile**	1	0	0	0	0	0	0	0
**seronegative**	**juvenile**	10	0	0	0	0	0	0	0
